# ROMO1 and CD47 in Cervical Cancer: Parallel but Independently Regulated

**DOI:** 10.3390/cimb48090862

**Published:** 2026-08-25

**Authors:** Angel Yordanov, Stoyan Kostov, Polina Damyanova Dimitrova, Ihsan Hasan, Eva Tsoneva

**Affiliations:** 1 Department of Gynaecological Oncology, Medical University Pleven, 5800 Pleven, Bulgaria; angel.jordanov@gmail.com; 2 Department of Gynecology, Hospital “Saint Anna”, Medical University—“Prof. Dr. Paraskev Stoyanov”, 9002 Varna, Bulgaria; 3 Research Institute, Medical University Pleven, 5800 Pleven, Bulgaria; 4 Department of General and Clinical Pathology, Heart and Brain Center of Clinical Excellence, 5800 Pleven, Bulgaria; 5 Department of Gynaecology and Pelvic Surgery, Hospital (MHAT), Vita 2, 1407 Sofia, Bulgaria; ihsan_hasanov@abv.bg; 6 Department of Gynaecology and Gynaecologic Oncology, Military Medical Academy, 1606 Sofia, Bulgaria

**Keywords:** cervical cancer, ROMO1, CD47, oxidative stress, immune evasion, immunohistochemistry

## Abstract

Reactive Oxygen Species Modulator 1 (ROMO1) and CD47 have both been implicated in cervical carcinogenesis, but their relationship within the same tumor has not been investigated. We retrospectively analyzed immunohistochemical H-scores for ROMO1 and CD47 in 221 invasive cervical carcinomas, including 205 tumors with evaluable expression of both markers, and examined their associations with clinicopathological characteristics. ROMO1 and CD47 expression did not correlate at the individual-tumor level (Spearman ρ = −0.08, *p* = 0.25; κ = −0.10). CD47 expression decreased significantly with increasing local tumor extent (ρ = −0.21, *p* = 0.002) and FIGO stage (*p* = 0.006), while ROMO1 showed a weaker, non-significant trend in the same direction. The ROMO1-high/CD47-high phenotype was observed in 15.7% of early-stage tumors but was absent in locally advanced (pT2–pT4) tumors (χ^2^ *p* = 0.001). These findings suggest that ROMO1 and CD47 are not directly co-regulated in cervical cancer, despite showing similar changes with local tumor progression. Their parallel expression patterns may therefore reflect separate responses to common factors involved in cervical carcinogenesis rather than a direct relationship between the two proteins. Further functional studies are needed to clarify the mechanisms underlying these findings.

## 1. Introduction

Cervical cancer remains one of the leading causes of cancer morbidity and mortality among women worldwide, particularly in regions with limited screening and vaccination coverage [1]. Persistent infection with high-risk human papillomavirus (HPV) types, principally HPV16 and HPV18, is the necessary—though not sufficient—driver of malignant transformation. The viral oncoproteins E6 and E7 inactivate p53 and pRb respectively, reprogram cellular metabolism toward aerobic glycolysis, and generate a state of chronic oxidative stress that is now recognized as a central, early feature of HPV-driven carcinogenesis [2].

Reactive Oxygen Species Modulator 1 (ROMO1) is a small inner-mitochondrial-membrane protein that regulates the production of mitochondrial ROS and, more recently, has been shown to form a non-selective cation channel that also functions as a redox sensor [3]. The clinical relevance of ROMO1 has been demonstrated in several malignancies. High ROMO1 expression has been associated with shorter disease-free and overall survival in non-small-cell lung cancer [4]. Similar findings have been reported in colorectal cancer, where increased ROMO1 expression was associated with poorer survival and a higher lymph-node ratio [5]. Increased ROMO1 expression has also been observed in higher-grade bladder tumors [6], while more recent studies have further supported its prognostic relevance in lung adenocarcinoma [7]. In glioblastoma, ROMO1 has additionally been shown to reprogram tumor-associated macrophages toward an immunosuppressive M2 phenotype, providing a plausible—though largely unexplored—mechanistic bridge between mitochondrial redox state and the anti-tumor immune response [8]. In cervical carcinogenesis specifically, ROMO1 has been proposed to act as an early, adaptive antioxidant buffer against HPV-oncoprotein-induced oxidative stress, with expression rising through preneoplastic (CIN) lesions and becoming heterogeneous and, in more advanced local disease, comparatively attenuated in invasive carcinoma [9]. The invasive carcinoma cohort analyzed for ROMO1 in that study (*n* = 205) is the same cohort included in the paired ROMO1/CD47 analysis presented here [9]. Accordingly, the previously reported ROMO1 and CD47 data [9,10] are included in the present study to enable direct within-tumor comparison of the two markers and analysis of their combined expression; the single-marker stage associations are therefore not considered independent or novel findings. CD47 is a ubiquitously expressed transmembrane immunoglobulin that engages SIRPα on macrophages to deliver a “don’t eat me” signal, thereby protecting tumor cells from phagocytic clearance by tumor-associated macrophages (TAMs) [10,11]. The clinical relevance of CD47 expression has been demonstrated across several solid malignancies. In colorectal cancer, increased CD47 expression has been associated with adverse clinicopathological features, including lymphatic and perineural invasion, higher nodal and AJCC stage, and poorer recurrence-free survival [12]. Similar findings have been reported in epithelial ovarian cancer, where high CD47 expression was associated with advanced FIGO stage, lymph-node metastasis, platinum resistance, and poorer prognosis [13]. More recently, high CD47 expression in breast cancer has been linked to distant metastasis, advanced TNM stage, and poorer overall survival [14]. In cervical cancer specifically, our group previously established the H-score as the most sensitive immunohistochemical method for quantifying CD47 expression and showed that high CD47 expression was significantly more frequent in early (pT1b) than in locally advanced (pT2a/pT2b) tumors and in FIGO stage I compared with stages II–III [10]; this pattern was subsequently replicated in a larger, partially overlapping cohort from the same institution, which additionally linked high CD47 expression to greater intratumoral macrophage infiltration [15]. A recent independent TCGA-based analysis likewise implicated CD47 (together with CD24) in an unfavorable cervical cancer phenotype, contingent on the local macrophage compartment [16]. Part of what follows reproduces rather than extends earlier work. The stage-related findings for ROMO1 derive from the same invasive carcinoma cohort previously reported in our ROMO1-focused study [9], while the associations of CD47 with pT and FIGO stage largely repeat our previous findings in an overlapping cohort [10]. These results are included to provide the necessary context for the paired-marker analysis. What is new here is the joint analysis of ROMO1 and CD47 within the same tumors, including their direct correlation and stage-related co-expression patterns.

There is a specific mechanistic reason to expect these two proteins to move together. ROS generated through ROMO1 activate NF-κB and thereby promote tumor-cell invasion [17], and NF-κB is among the redox-sensitive transcriptional regulators reported to control CD47 expression [18]. More broadly, ROS do not act only inside the cell that produces them: they diffuse through the tumor microenvironment and reach the surrounding immune compartment, where sustained oxidative stress polarizes tumor-associated macrophages toward immunosuppressive phenotypes, blunts T-cell function and alters the expression of immune-checkpoint molecules [19,20]. If a single ROS-driven program governed both arms, a redox regulator such as ROMO1 and a checkpoint such as CD47 should rise and fall together. Whether they actually do, within the same tumor, is what the present study set out to test.

Several considerations guided the decision to study these two markers together rather than to screen a broad panel of redox- or immune-related proteins. Both have already been characterized in cervical tissue by our group with the same H-score approach and in overlapping material, so a joint analysis could be performed on a consistent, directly comparable footing. Each also represents one end of a biologically plausible connection between HPV-driven oxidative stress and immune escape: ROMO1 as the marker we have used to follow the mitochondrial redox response, and CD47 as the dominant checkpoint shielding tumor cells from macrophage attack. A link between the two is not merely speculative, since ROMO1 overexpression has been shown to push tumor-associated macrophages toward an immunosuppressive M2 state in glioblastoma [8], which makes it reasonable to ask whether an analogous redox-to-immune-escape relay might operate through CD47 in cervical cancer. On these grounds, ROMO1 and CD47 seemed the natural pair to test whether redox adaptation and immune evasion are regulated together.

Each marker thus reflects an adaptive response that is prominent early in local tumor evolution and appears to wane with progression. We use the term stress–escape axis as shorthand for the hypothesized coupling between the intracellular oxidative-stress response (ROMO1) and extracellular, macrophage-directed immune evasion (CD47). The central question is whether the two are regulated together within the same tumor cell population—perhaps through a shared HPV-oncoprotein signal that simultaneously buffers oxidative stress and confers immune evasion—or whether they are triggered in parallel but independently. To our knowledge no previous study has looked at ROMO1 and CD47 together in cervical cancer. We addressed this using a single-institution immunohistochemical cohort, asking first whether ROMO1 and CD47 expression correlate within individual tumors, and second whether their combined expression relates to local tumor stage in a way that matches or departs from the behavior each marker shows on its own.

Part of what follows reproduces rather than extends earlier work. The associations of CD47 with pT and FIGO stage (Section 3.3) largely repeat what we reported in a smaller, partly overlapping cohort from the same institution [10] and what was later confirmed in a larger series [15]; we include them chiefly to confirm that the present dataset behaves as expected. What is new here is the joint treatment of the two markers: this is the first analysis to test directly, rather than assume, whether mitochondrial redox adaptation and macrophage-mediated immune evasion move together at the level of the individual tumor; it yields an explicit negative result for that coupling. It also uncovers a stage-restricted ROMO1/CD47 co-expression pattern, confined to early and intermediate disease and absent in locally advanced tumors, that neither marker reveals on its own.

## 2. Materials and Methods

### 2.1. Patients and Specimens

This retrospective, single-institution study included paraffin-embedded tumor tissue from patients with histologically confirmed invasive cervical carcinoma. Clinicopathological data—histological subtype (squamous cell carcinoma [SCC], adenocarcinoma [AC], adenosquamous carcinoma [ASC]), pathological TNM stage, FIGO stage, tumor grade, and lymphovascular space invasion (LVSI)—were retrieved from the institutional pathology database. Ethical committee approval was obtained (number 656/29 June 2021) to investigate the role of ROMO1 in paraffin-embedded tumor tissue from those patients. Patients’ clinico-morphological information was retrieved from the Department’s electronic database. Data were de-identified prior to statistical analysis; direct identifiers (name, national identification number, exact date of birth) were removed and are not reported anywhere in this manuscript.

#### Data Quality Assurance

Prior to analysis, the source database was screened for transcription artifacts. Two categorical fields contained recognizable errors introduced during data entry/extraction (a malformed pT sub-stage label and a single mislabeled LVSI value); pT sub-stage was therefore re-derived programmatically from the combined pTN stage field, and the affected LVSI value was corrected. For analyses stratified by local tumor extent, pT sub-stages were grouped into three clinically coherent categories: early (pT1a–pT1b1), intermediate (pT1b2–pT1b3) and locally advanced (pT2a–pT4). Missing data handling: Grade and LVSI information were unavailable for a subset of patients. Because the missingness arose from incomplete historical pathology records rather than study-related exclusions, analyses involving these variables were performed on available cases only (complete-case analysis), without imputation. The proportion of missing values is reported in the Section 3.

### 2.2. Immunohistochemistry and Scoring

CD47 and ROMO1 expression were assessed by immunohistochemistry using the H-score. For each patient, a representative hematoxylin–eosin-stained slide was selected, and the corresponding formalin-fixed, paraffin-embedded tumor block was used for immunohistochemical analysis. Sections of 3 μm thickness were prepared.

CD47 staining was performed as previously described by our group [10], using an anti-CD47 antibody (clone SP279, rabbit monoclonal, dilution 1:100; Abcam, Cambridge, UK). ROMO1 staining was performed according to our previously reported protocol [9], using an anti-ROMO1 antibody (clone OTI2C12, mouse monoclonal, dilution 1:150; Abcam, Cambridge, UK). Immunohistochemistry was performed using the EnVision™ FLEX detection system (Agilent, Santa Clara, CA, USA) and Autostainer Link 48 platform (DAKO) (Agilent, Santa Clara, CA, USA), with heat-mediated antigen retrieval in citrate buffer (pH 6). Appropriate external positive controls were included in each staining run.

Expression was evaluated semi-quantitatively using the H-score. The entire tumor section was examined at low and high magnification, taking into account both staining intensity and the percentage of positive viable tumor cells. Immunohistochemical slides were independently evaluated by two pathologists using the same predefined H-score criteria. Staining intensity was scored as absent (0), weak (1+), moderate (2+), or strong (3+). The H-score was calculated as (% of weakly stained cells × 1) + (% of moderately stained cells × 2) + (% of strongly stained cells × 3), giving a final score from 0 to 300.

Previously established marker-specific cut-offs were used to categorize expression [9,11]. CD47 expression was classified as negative (H-score = 0), low (H-score > 0 to ≤74), or high (H-score > 74) [11]. ROMO1 expression was classified as negative (H-score = 0), low (H-score > 0 to ≤68.40), or high (H-score > 68.40) [9]. These negative, low, and high categories refer to the classification of the calculated H-score and should not be confused with the four staining-intensity levels used to calculate the score.

**Figure 1:**
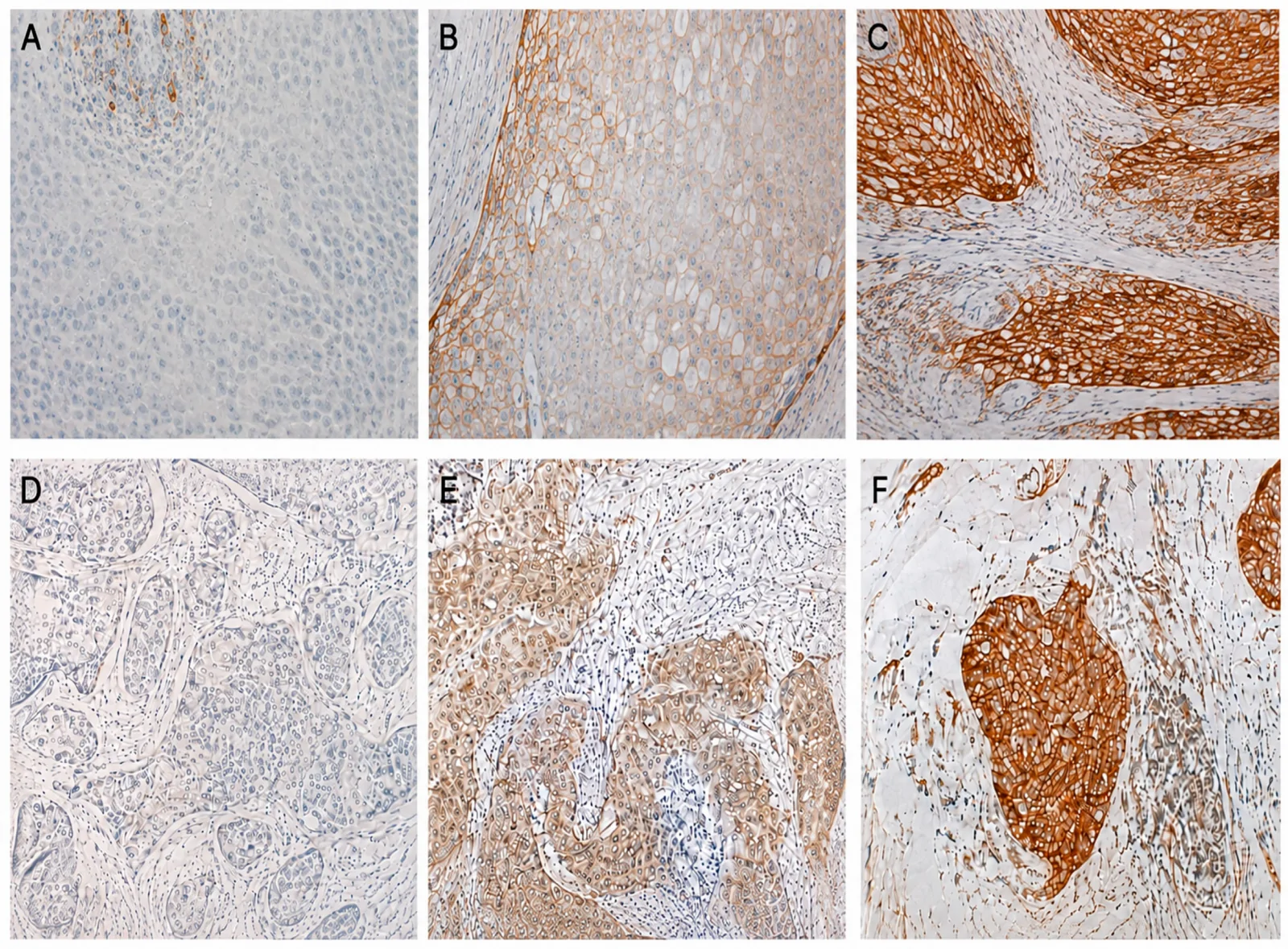
Representative immunohistochemical expression of CD47 and ROMO1 in cervical carcinoma. Representative CD47 immunohistochemical staining showing negative (**A**), low (**B**), and high (**C**) expression, and representative ROMO1 immunohistochemical staining showing negative (**D**), low (**E**), and high (**F**) expression. CD47 expression was categorized as negative (H-score = 0), low (H-score > 0 and ≤74), or high (H-score > 74) [11]. ROMO1 expression was categorized as negative (H-score = 0), low (H-score > 0 and ≤68.40), or high (H-score > 68.40) [9]. Representative images were adapted from Refs. [9,11].

For statistical analyses, these categories were coded as 0 (negative), 1 (low), and 2 (high). For the co-expression analysis, expression was further dichotomized as high versus low/negative (category 2 vs. categories 0–1). Representative immunohistochemical staining patterns for ROMO1 and CD47 are shown in Figure 1.

### 2.3. Statistical Analysis

The association between ROMO1 and CD47 H-score categories (ordinal, 0/1/2) was assessed with Spearman’s rank correlation, Kendall’s τ, and a χ^2^ test of the 3 × 3 contingency table. Agreement between the dichotomized markers (high versus low/negative) was examined with Fisher’s exact test and Cohen’s κ. Each marker was related to the categorical clinicopathological variables (histological subtype, N status, grade, LVSI, FIGO stage) by χ^2^ test—with Fisher’s exact test substituted where more than 20% of expected cell counts fell below five—and to the ordered variables (T-stage group, FIGO stage) by Spearman correlation, treating the ordered category as a rank so as to test for a monotonic trend rather than an omnibus difference. Given that only one patient had FIGO stage IV disease, the FIGO-stage trend analyses were repeated after excluding this case as a sensitivity analysis. A three-level co-expression phenotype (concordant-high, discordant, concordant-low/negative) was derived from the dichotomized markers and tested against T-stage group by χ^2^. To confirm that this phenotype did not depend on the particular dichotomization threshold, an unsupervised four-cluster solution (k-means, checked against hierarchical clustering) was fitted to the joint ROMO1/CD47 H-score data, and cluster membership was cross-tabulated with histological subtype and—within SCC—with T-stage group, N status, grade, LVSI, FIGO stage and age. Finally, multivariable logistic regression tested whether ROMO1 H-score predicted CD47-high status once T-stage group and histological subtype were accounted for in order to see whether the null bivariate correlation held after adjusting for the association of both markers with local stage. A two-sided *p* < 0.05 was taken as significant; because the analysis was exploratory, no correction for multiple comparisons was applied, and this is noted among the limitations. Analyses were carried out in Python (version 3.12.3) (pandas, SciPy).

Sensitivity analysis (minimum detectable effect). To aid interpretation of the primary null finding, the minimum detectable correlation was estimated for the paired-marker cohort. Assuming a two-sided significance level of α = 0.05 and 80% statistical power, the available sample size (*n* = 205 paired ROMO1/CD47 observations) was sufficient to detect correlations of approximately |ρ| ≥ 0.20. Accordingly, the study was adequately powered to exclude moderate or stronger associations between ROMO1 and CD47 expression, whereas very weak correlations cannot be excluded.

## 3. Results

### 3.1. Cohort Characteristics

Of the 226 patients in the database, five had neither marker successfully assessed and were excluded, leaving an analytic cohort of 221 patients (mean age 51.5 ± 11.9 years, range 25–82); 205 of these had both markers and form the set for all paired analyses. SCC accounted for 68.3% of cases (*n* = 151), AC for 22.2% (*n* = 49) and ASC for 9.5% (*n* = 21). Distribution by FIGO stage was: stage I 63.3% (*n* = 140), stage II 9.0% (*n* = 20), stage III 27.1% (*n* = 60), and stage IV 0.5% (*n* = 1). By the grouped local (pT) stage used in this analysis, 32.6% of tumors (*n* = 72) were early (pT1a–pT1b1), 48.0% (*n* = 106) intermediate (pT1b2–pT1b3) and 19.5% (*n* = 43) locally advanced (pT2a–pT4). Nodal involvement (N1) was present in 27.6% (*n* = 61). Grade and LVSI data were incomplete for a subset of patients (18.1% and 27.1% missing, respectively) and are reported on available cases (Table 1).

### 3.2. ROMO1 and CD47 Expression Do Not Correlate at the Individual-Tumor Level

**Figure 2:**
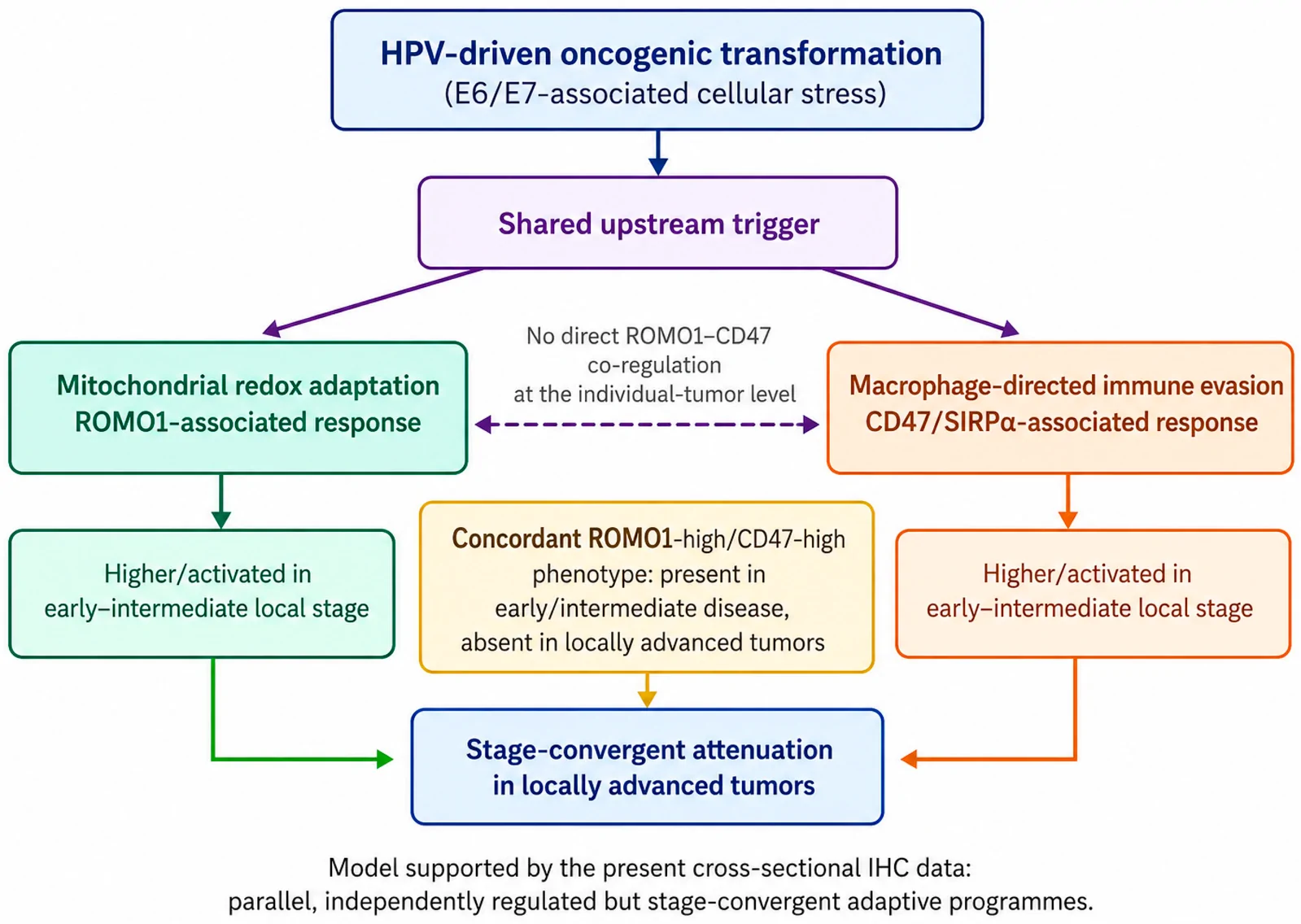
Proposed conceptual model. HPV-driven oncogenic transformation is hypothesized as a shared upstream trigger for two mechanistically distinct adaptive programmes—mitochondrial redox adaptation (ROMO1) and macrophage-directed immune evasion (CD47/SIRPα)—which were not directly co-regulated at the individual-tumor level (dashed arrow), but each showed higher activity in early/intermediate disease and attenuation in locally advanced tumors, converging on a stage-restricted concordant ROMO1-high/CD47-high phenotype that was absent in locally advanced disease. The shared-upstream-trigger node is a working hypothesis consistent with—, but not directly tested by—, the present cross-sectional data, which lacked HPV E6/E7 transcript measurements (see Section Limitations).

Among 205 tumors with paired ROMO1 and CD47 H-score data, no significant correlation was observed between the two markers (Spearman ρ = −0.081, *p* = 0.249; Kendall’s τ = −0.075, *p* = 0.248; χ^2^ = 4.24, df = 4, *p* = 0.374; Table 2). When both markers were dichotomized (H-score = 2 [“high”] vs. 0–1 [“low/negative”]), the association remained non-significant (Fisher’s exact *p* = 0.113, OR = 0.61), and agreement between the two markers was effectively absent (Cohen’s κ = −0.10). These results indicate that, within an individual tumor, high ROMO1 expression does not predict high CD47 expression, or vice versa. Visualization of Pearson residuals from the contingency table confirmed the absence of any cell with substantial deviation from independence (all |residuals| < 2; Figure 2).

Because ROMO1 and CD47 each associate with histological subtype (Section 3.3), the ROMO1–CD47 correlation was additionally examined within each histotype separately to test whether the overall null result masked a subtype-specific relationship. No significant correlation was found within SCC (*n* = 137; Spearman ρ = −0.008, *p* = 0.928), the largest and most homogeneous subgroup. A non-significant negative trend was observed within AC (*n* = 49; ρ = −0.274, *p* = 0.057) and no association was found within ASC (*n* = 19; ρ = −0.068, *p* = 0.782). The absence of coupling is therefore not attributable to a masked, histotype-specific relationship; if anything, the only subgroup approaching significance showed a trend in the negative, not positive, direction.

### 3.3. Each Marker Shows an Independent Association with Local Tumor Stage

**Figure 3:**
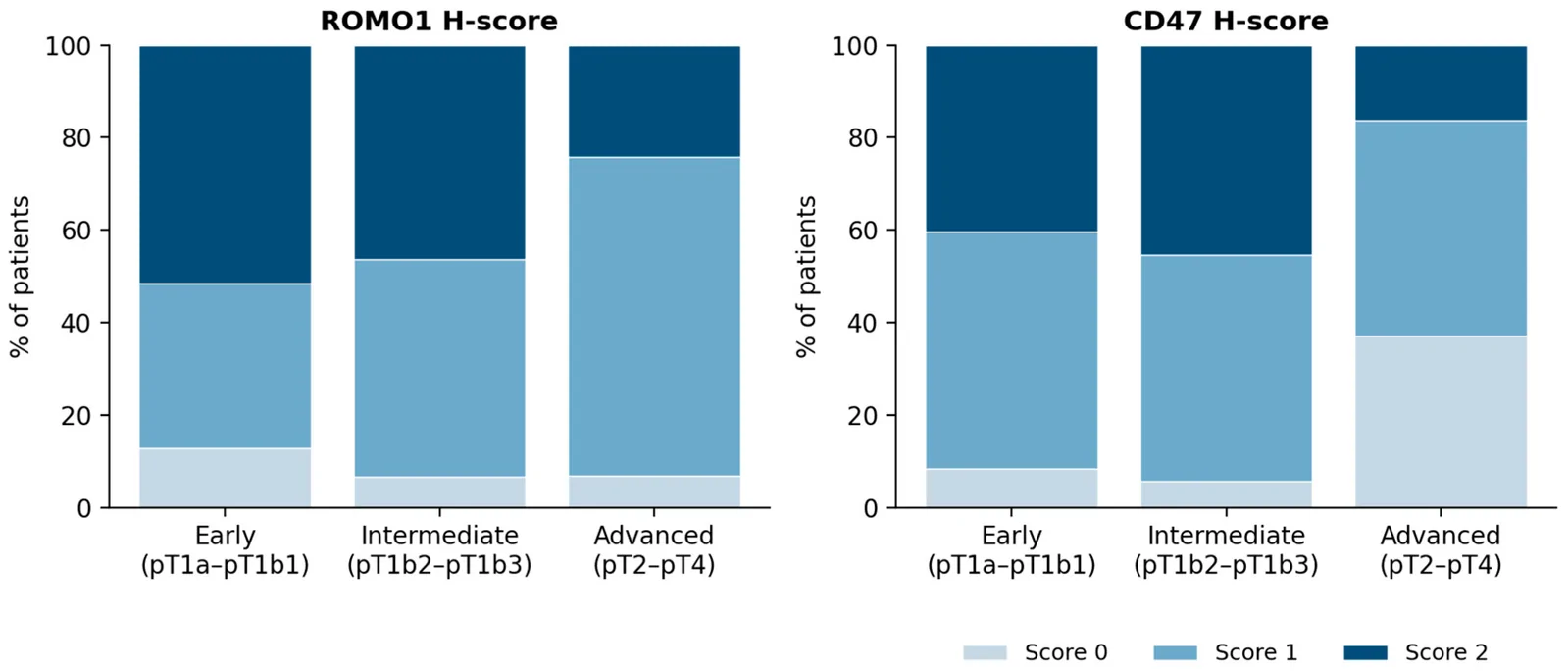
Distribution of ROMO1 (**left**) and CD47 (**right**) H-score categories (0 = negative, 1 = low, 2 = high) across grouped local (pT) tumor stage. Both markers show a decline in the high-expression fraction in locally advanced (pT2–pT4) tumors relative to early/intermediate disease; the trend reaches statistical significance for CD47 and represents a weaker, non-significant tendency for ROMO1 in this cohort.

CD47 expression decreased with increasing local tumor extent. CD47-negative tumors accounted for 37.2% of locally advanced cases compared with only 8.1% of early-stage tumors, while high CD47 expression decreased to 16.3% in the locally advanced group (χ^2^ *p* < 0.0001; ordinal trend ρ = −0.205, *p* = 0.002). CD47 expression also decreased with increasing FIGO stage (ρ = −0.183, *p* = 0.006), whereas no significant associations were found with N status, grade, or LVSI (all *p* > 0.10). The associations with local tumor extent and FIGO stage, together with the lack of association with nodal status, are consistent with our previous findings [10]. ROMO1 showed a similar, although weaker, pattern across the T-stage groups (ρ = −0.102, *p* = 0.147). ROMO1 expression was significantly associated with histological subtype (χ^2^ *p* = 0.021) and with T-stage group in the categorical analysis (χ^2^ *p* = 0.034), but not with N status, grade, LVSI, or FIGO stage (all *p* > 0.15). Figure 3 shows the distribution of both markers across T-stage groups.

Given that only one patient had FIGO stage IV disease, the ordinal trend analyses were repeated after excluding this case. The results were essentially unchanged: the association between CD47 expression and FIGO stage remained significant (ρ = −0.181, *p* = 0.007), whereas the association between ROMO1 expression and FIGO stage remained non-significant (ρ = −0.109, *p* = 0.121). Thus, the single FIGO IV case did not materially influence the observed stage-related trends.

### 3.4. A Concordant ROMO1-High/CD47-High Phenotype Is Absent in Locally Advanced Disease

**Figure 4:**
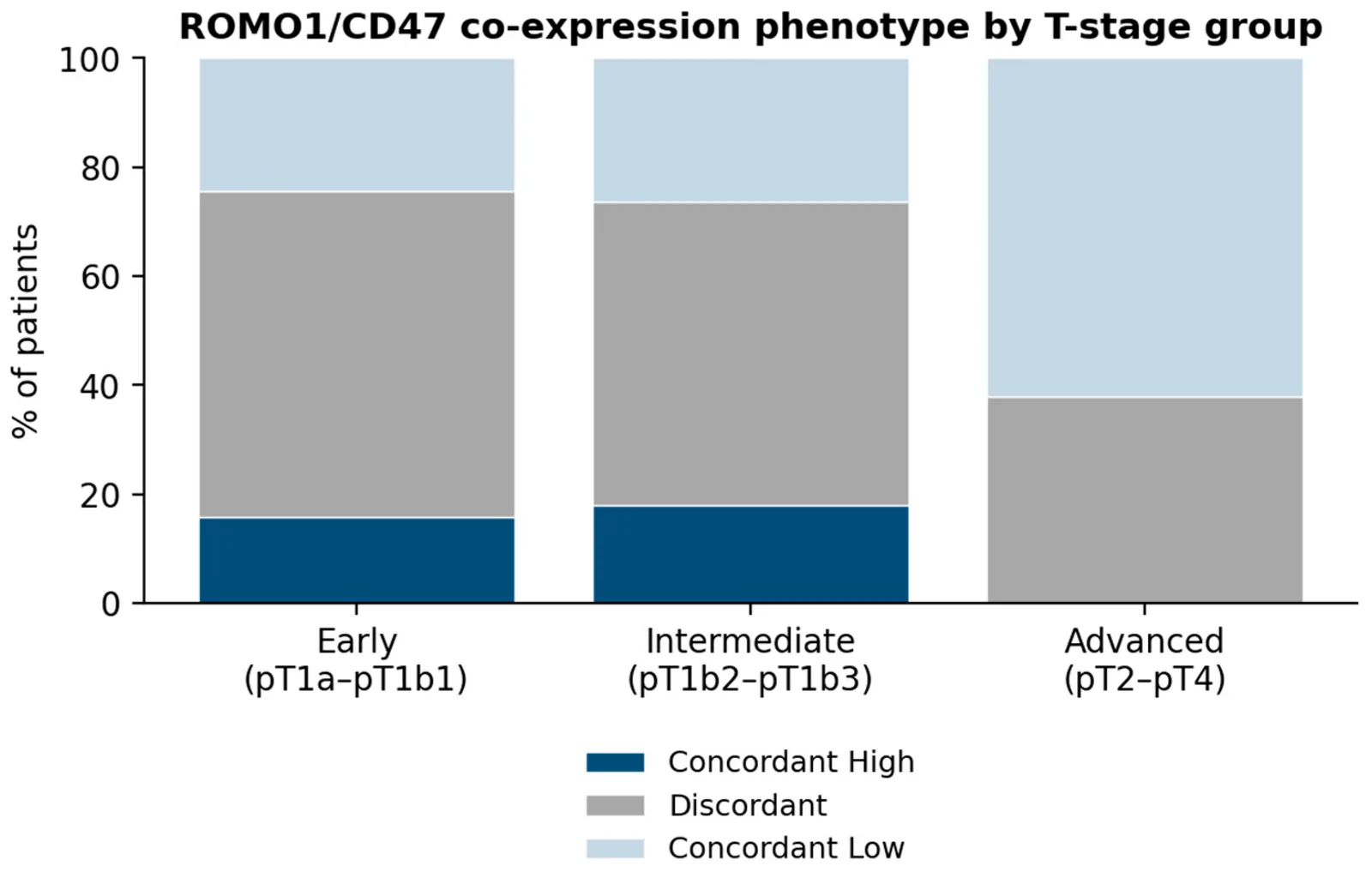
Distribution of the ROMO1/CD47 co-expression phenotype across T-stage groups. The concordant ROMO1-high/CD47-high phenotype (dark blue) is present in a minority of early- and intermediate-stage tumors but is entirely absent among locally advanced (pT2–pT4) tumors (χ^2^ *p* = 0.0013).

Because the two markers did not correlate directly, we examined whether their joint (rather than pairwise-correlated) expression pattern nonetheless tracked tumor stage. Tumors were classified as concordant-high (both markers dichotomized “high”), concordant-low/negative (both “low/negative”), or discordant (one high, one low/negative). Overall, 14.6% of tumors were concordant-high, 30.7% concordant-low/negative and 54.6% discordant. This phenotype distribution differed significantly across T-stage groups (χ^2^ *p* = 0.0013): the concordant-high phenotype was present in 15.7% of early-stage and 17.8% of intermediate-stage tumors, but in 0% (0/29) of locally advanced (pT2–pT4) tumors within the paired-marker cohort (*n* = 205), among which the concordant-low/negative phenotype instead predominated (62.1%; Figure 4).

### 3.5. Sensitivity Analysis: Unsupervised Expression Clusters and Multivariable Adjustment

To verify that the co-expression phenotype described above was not an artifact of the specific H-score dichotomization threshold, an unsupervised four-cluster solution was fitted to the joint ROMO1/CD47 H-score data (k-means, confirmed with hierarchical clustering; 100% agreement between the two methods for three of four clusters and >85% overall). The resulting clusters corresponded closely to the phenotype groups already described: one cluster (*n* = 30) comprised exclusively concordant ROMO1-high/CD47-high tumors, one (*n* = 63) comprised exclusively concordant low/negative tumors, and the remaining tumors separated into two discordant sub-clusters distinguished by which marker was dominant (CD47-high/ROMO1-low, *n* = 50; ROMO1-high/CD47-low, *n* = 62). Cluster membership differed significantly by histological subtype, both when SCC was compared with non-SCC tumors combined (χ^2^ *p* = 0.0007) and across the three histotypes separately (χ^2^ *p* = 0.0045).

Restricting this cluster analysis to SCC alone (*n* = 137, the largest and most homogeneous histotype in the cohort) showed that cluster membership was significantly associated with T-stage group (χ^2^ *p* = 0.0068) and, more weakly, with FIGO stage (χ^2^ *p* = 0.045), but not with N status, grade, LVSI, or age (all *p* ≥ 0.48; Kruskal–Wallis *p* = 0.82 for age). This indicates that, within the most common histotype, the joint ROMO1/CD47 expression pattern tracks specifically with local anatomical tumor extent rather than with nodal spread, differentiation, lymphovascular invasion or patient age—mirroring, at a finer resolution, the T-stage-restricted pattern already described for the three-level phenotype in Section 3.4.

Because both ROMO1 and CD47 independently associate with T-stage and histotype (Section 3.3), a multivariable logistic regression was used to test whether ROMO1 predicted CD47-high status after adjusting for these shared associations, rather than relying on the unadjusted bivariate correlation alone. In this model (*n* = 205; CD47-high vs. CD47-low/negative as the outcome), both T-stage group and histological subtype remained independently associated with CD47-high status (early vs. advanced T-stage: OR = 7.46, 95% CI 2.15–25.91; SCC vs. AC: OR = 4.57, 95% CI 1.87–11.19), consistent with Section 3.3. ROMO1 H-score showed a borderline independent inverse association with CD47-high status after this adjustment (OR = 0.62 per H-score category, 95% CI 0.39–1.00, *p* = 0.051; Table 3)—that is, once local stage and histotype are accounted for, higher ROMO1 was weakly associated with lower, not higher, odds of high CD47 expression. This estimate did not reach conventional statistical significance and is presented as a hypothesis-generating signal rather than a confirmed independent association; it is, notably, opposite in direction to what a model of coordinated up-regulation would predict.

### 3.6. Sensitivity to Classification of CD47-Negative Tumors

Because the primary result is null, we examined the extent to which it depends on the small number of tumors scored CD47-negative, the category at the boundary of the semi-quantitative scale where classification is least certain and where few cases carry disproportionate weight in a rank correlation. CD47-negative tumors were uncommon (*n* = 21 of 205). Excluding them left the conclusion unchanged in direction and significance but shifted the point estimate (*n* = 184, Spearman ρ = −0.138, *p* = 0.062). The principal finding, that ROMO1 and CD47 are not positively co-expressed, therefore holds under both specifications; the precise magnitude of the weak negative tendency does not, and should not be over-interpreted.

## 4. Discussion

The CD47-alone findings in Section 3.3 reproduce our own and other groups’ earlier cervical cancer studies [11,15,16] and are best read as a check that the present cohort and analysis behave as they should, not as new observations. The novel elements are the joint analysis of ROMO1 and CD47 in the same tumors (Section 3.2); the negative result for their correlation; and the stage-restricted co-expression pattern of Section 3.4, which is the main new finding.

The null correlation is best judged against what the cohort could realistically detect. With the available sample, the study was sensitive to moderate correlations but not to very weak ones, so the data argue against ROMO1–CD47 co-regulation of moderate or greater magnitude while leaving subtler effects unresolved.

Three points stand out from these results.

First, ROMO1 and CD47 did not correlate within individual tumors, and this held across every test we applied (Spearman, Kendall, χ^2^, the dichotomized Fisher’s exact test, and Cohen’s κ). Contrary to our starting assumption, the strength of the ROMO1-marked redox response inside a given tumor tells us nothing about the strength of its CD47-mediated “don’t eat me” signal, and the two stains cannot stand in for one another.

Second, each marker nonetheless behaved similarly with respect to local stage. CD47 was clearly lower in locally advanced (pT2–pT4) than in early or intermediate tumors, again matching our earlier report in a smaller cohort [10] and the later confirmation in a larger series [15], both of which placed CD47 highest in early pT1b disease and lower in pT2a/pT2b tumors. ROMO1 followed the same direction more weakly and without reaching significance, and was additionally linked to histological subtype—in keeping with earlier tissue observations of its heterogeneity across cervical histotypes [9]. Our earlier study in a smaller cohort of 75 patients showed significantly higher ROMO1 expression in FIGO stage I than in stages II and III [21]. In the present larger cohort, ROMO1 expression was again higher in earlier-stage tumors, but the association with FIGO stage did not reach statistical significance [9]. Thus, although the significant FIGO-stage association observed in the smaller series was not reproduced, both studies showed the same overall pattern of higher ROMO1 expression in earlier disease and lower expression with advancing stage [9,21]. That the CD47 pattern reproduces so closely is reassuring for the reliability of the present cohort and its cleaning.

Third, looking at the two markers jointly exposed something that neither showed alone: tumors high in both ROMO1 and CD47 clustered in early and intermediate disease and were absent among locally advanced cases. The pattern did not hinge on how we set the threshold, since an unsupervised four-cluster solution recovered the same groupings, and within SCC the clusters tracked T-stage (and, more weakly, FIGO stage) but not grade, LVSI, nodal status, or age, pointing to local anatomical extent rather than to aggressiveness in general. The multivariable model added a further wrinkle: with T-stage and histotype held constant, higher ROMO1 was associated, at borderline significance, with lower odds of CD47-high status (OR = 0.62, *p* = 0.051)—the reverse of what coordinated up-regulation would produce, and visible only after adjusting for the stage effect shared by both markers. Taken together, the concordant ROMO1-high/CD47-high state looks less like the arithmetic sum of two stage-linked markers and more like a specific, stage-restricted condition, most plausibly reflecting a shared upstream trigger acting on both programs at once rather than one marker driving the other. This remains a hypothesis to be tested in an independent, adequately powered cohort.

The contrast is laid out in Table 4: across the cohort as a whole, ROMO1 and CD47 move almost in step—yet within individual tumors they show no coupling at all. We read this as two parallel rather than sequential adaptations—both potentially influenced by HPV-associated cellular stress, although through distinct downstream pathways [2,22]. HPV-driven oxidative stress is increasingly recognized as one component of a much broader network involving metabolic rewiring, chronic inflammation, and immune remodeling during cervical carcinogenesis, providing several potential upstream mechanisms capable of affecting ROMO1 and CD47 independently [23].

A plausible biological explanation for the absence of direct correlation is that ROMO1 and CD47 are governed by distinct upstream regulatory circuits that happen to share the same ultimate trigger (HPV oncoprotein-driven cellular stress) without being causally linked to one another [2]. ROMO1 induction is understood mechanistically as an intrinsic mitochondrial response to oxidative stress and is linked—in other tumor types—to NF-κB-dependent invasion signaling [3,17] and, in glioblastoma, to macrophage reprogramming toward an M2, immunosuppressive phenotype via mTORC1 signaling in the macrophages themselves rather than in tumor cells [8]. This concept is also consistent with broader evidence that oxidative stress remodels the tumor immune microenvironment through multiple independent signaling pathways rather than a single linear cascade, influencing macrophage polarization, T-cell function, and immune-checkpoint regulation [24,25].

CD47 up-regulation, by contrast, is more directly tied to interactions with the local immune/macrophage compartment and to signals such as interferon and NF-κB pathways acting on the SIRPα axis [11,18,22,26]. These are biologically adjacent but mechanistically separable pathways, consistent with the pattern of statistical independence combined with population-level convergence observed here.

These findings should be interpreted in the context of the broader two-phase model proposed for ROMO1 in HPV-associated cervical carcinogenesis, in which an early, adaptive redox response is progressively lost as tumors advance locally [2,9,21]. The present data extend that model by showing that CD47-mediated immune evasion follows an analogous early-activation/late-attenuation trajectory, but do not support the stronger claim that the two are mechanistically coupled at the level of a single, unified adaptive program within the tumor cell. Direct testing of this relationship—for example, whether HPV E6/E7 silencing or induced oxidative stress in cervical cell lines (SiHa, CaSki, HeLa, and the W12 episomal/integrated progression model) alter CD47 expression independently of ROMO1, or vice versa—would help establish whether the observed independence reflects genuine biological separation or is a limitation of a cross-sectional, semi-quantitative immunohistochemical design.

The relationships described above can be summarized in a proposed conceptual model (Figure 4). The model distinguishes the components that are directly supported by the present cross-sectional analysis—parallel, stage-dependent attenuation of both markers; absence of direct co-regulation at the individual-tumor level; and stage-restricted convergence of the joint phenotype in early/intermediate disease—from the proposed shared upstream trigger, which reflects a working hypothesis motivated by the known biology of HPV oncoproteins rather than a relationship directly tested in this dataset.

### Limitations

- The retrospective, single-institution cohort; findings require external replication.
- The FIGO-stage groups were unevenly represented, with particularly small numbers of stage II and IV cases, limiting stage-specific comparisons. However, the exclusion of the single FIGO IV case in a sensitivity analysis did not materially change the observed FIGO-stage trends.
- Semi-quantitative immunohistochemical scoring is subject to inter-observer variability, although a previously validated H-score cut-off was used [10].
- Missing data for grade (18.1%) and LVSI (27.1%) reduce power for these subgroup analyses.
- The cross-sectional design without linked survival/recurrence outcome data. Outcome-based (survival) analysis was not attempted in the present study because national treatment guidelines for cervical cancer changed during the recruitment period, so patients in this cohort received heterogeneous primary treatment (surgery alone, surgery with adjuvant (chemo)radiation, or primary chemoradiation) depending on diagnosis date rather than tumor biology alone; comparing survival across such a guideline-driven, treatment-heterogeneous cohort without adequate stratification would risk conflating treatment-era effects with any biomarker-related prognostic signal. The prognostic value of the joint ROMO1/CD47 phenotype should therefore be assessed in a future, treatment-stratified or treatment-homogeneous cohort with adequate follow-up.
- No correction for multiple comparisons was applied; results should be regarded as hypothesis-generating, particularly the co-expression phenotype finding.
- HPV genotype and E6/E7 transcript levels were not available, precluding direct testing of the proposed common upstream trigger.

**Figure 5:**
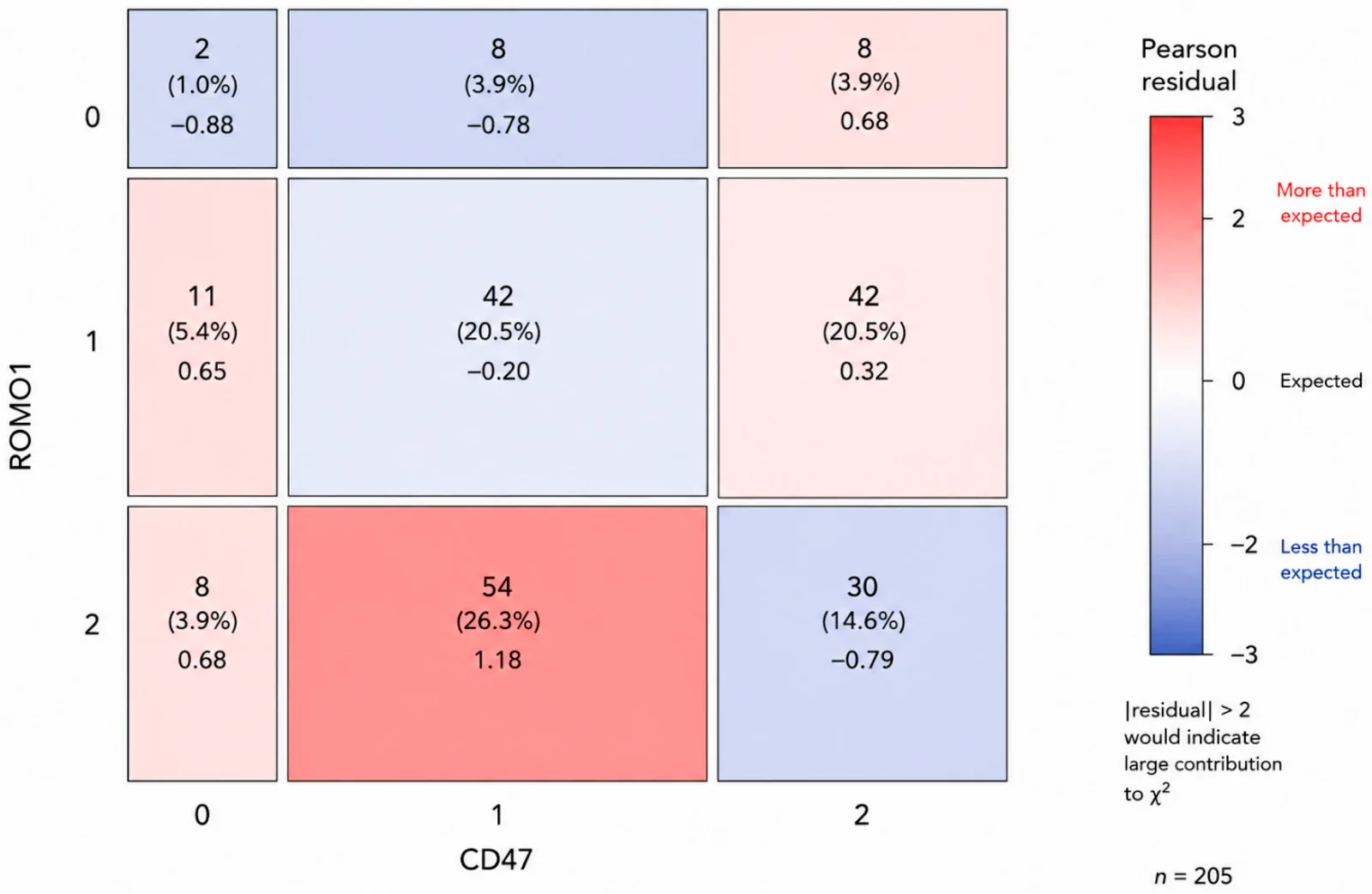
Mosaic plot showing the contingency table of ROMO1 and CD47 H-score categories (*n* = 205). Rectangle area is proportional to the number of tumors in each category. Color indicates the Pearson residual from the χ^2^ test of independence, with red denoting more observations than expected under independence and blue fewer observations than expected. No cell exceeded an absolute Pearson residual of 2, indicating that no individual cell contributed substantially to the overall χ^2^ statistic (χ^2^ = 4.24, df = 4, *p* = 0.374).

Although the paired-marker cohort was sufficiently powered to detect moderate correlations (approximately |ρ| ≥ 0.20), weaker associations cannot be excluded. Consequently, the present findings should be interpreted as evidence against moderate or strong ROMO1–CD47 coupling rather than definitive evidence for complete biological independence (Figure 5).

## 5. Conclusions

Within this cohort, ROMO1 and CD47 were uncorrelated at the level of the individual tumor, so the two markers of mitochondrial redox adaptation and macrophage-mediated immune evasion do not appear to be driven by a single shared program inside the tumor cell. Both, however, declined in parallel with local stage, and their joint high expression was confined to early and intermediate disease. Rather than confirming a unified redox–immune-evasion axis in HPV-associated cervical carcinogenesis, the data point to two independently regulated but stage-convergent responses. Cell-line studies will be needed to work out the causal relationships behind this pattern.

## Figures and Tables

**Table 1:** Baseline characteristics of the analytic cohort (*n* = 221).

Characteristic	Category	*n* (%)
Age (years)	Mean ± SD (range)	51.5 ± 11.9 (25–82)
Histological subtype	SCC	151 (68.3%)
	AC	49 (22.2%)
	ASC	21 (9.5%)
Local (pT) stage group *	Early (pT1a–pT1b1)	72 (32.6%)
	Intermediate (pT1b2–pT1b3)	106 (48.0%)
	Advanced (pT2a–pT4)	43 (19.5%)
N status	N0	160 (72.4%)
	N1	61 (27.6%)
FIGO stage	I	140 (63.3%)
	II	20 (9.0%)
	III	60 (27.1%)
	IV	1 (0.5%)
Grade (*n* = 181 available)	G1/G2/G3	50 (27.6%)/85 (47.0%)/46 (25.4%)
LVSI (*n* = 161 available)	Yes/No	33 (20.5%)/128 (79.5%)

* Microinvasive carcinomas (pT1a; *n* = 5) were grouped within the Early (pT1a–pT1b1) category.

**Table 2:** Cross-tabulation of ROMO1 and CD47 H-score categories (χ^2^ = 4.24, df = 4, *p* = 0.374; Spearman ρ = −0.081, *p* = 0.249).

ROMO1 H-Score	CD47 = 0 (*n*)	CD47 = 1 (*n*)	CD47 = 2 (*n*)	Row Total
0 (negative)	2	8	8	18
1 (low)	11	42	42	95
2 (high)	8	54	30	92
Column total	21	104	80	205

**Table 3:** Multivariable logistic regression for CD47-high status (*n* = 205); reference categories: T-stage Advanced (pT2–pT4), histotype AC.

Predictor	OR	95% CI	*p*
ROMO1 H-score (per category)	0.62	0.39–1.00	0.051
T-stage: Early vs. Advanced	7.46	2.15–25.91	0.002
T-stage: Intermediate vs. Advanced	5.25	1.65–16.74	0.005
Histotype: SCC vs. AC	4.57	1.87–11.19	0.001
Histotype: ASC vs. AC	1.68	0.46–6.15	0.435

**Table 4:** Comparative summary of ROMO1 and CD47 behavior in this cohort: similar population-level trajectory across tumor stage, but no evidence of coordinated regulation at the individual-tumor level.

Characteristic	ROMO1	CD47
Declines with local (T-stage) progression	Yes (trend, ns in full cohort)	Yes (significant)
Highest in early/intermediate disease	Yes	Yes
Significant individual-tumor correlation with the other marker	No	No
Correlation with the other marker within SCC specifically	No (ρ = −0.008, *p* = 0.928)	No (ρ = −0.008, *p* = 0.928)
Evidence of a shared single regulatory mechanism	Not demonstrated	Not demonstrated

## Data Availability

The de-identified dataset supporting the findings of this study is available from the corresponding author upon reasonable request.
